# Predictors of inadequate minimum acceptable diet among infants and young children in Ethiopia: a multilevel logistic regression analysis

**DOI:** 10.3389/fpubh.2025.1464008

**Published:** 2025-05-14

**Authors:** Muluhabt Alene Assfaw, Dereje Tesfaye, Haile Mekonnen Fenta, Wondaya Fenta Zewdia, Bisratgebriel Tesfaye Muchie, Daniel Asmelash

**Affiliations:** ^1^Department of Statistics, College of Science, Bahir Dar University, Bahir Dar, Ethiopia; ^2^College of Medicine and Health Sciences, Mizan-Tepi University, Mizan-Aman, Ethiopia

**Keywords:** determinants, minimum acceptable diet, minimal dietary diversity, minimum meal frequency dietary diversity, multilevel analysis, infants, children, Ethiopia

## Abstract

**Background:**

Inadequate minimum acceptable diet is the cause of poor physical and mental development and poses a significant burden among infants and young children aged between 6 and 23 months. The primary purpose of this study was to determine the factors contributing to inadequate minimum acceptable diet among infants and young children in Ethiopia.

**Method:**

The 2019 Ethiopian Mini Demographic and Health Survey dataset, with 1,463 weighted samples of children aged 6–23 months, was used. Data management was performed using STATA version 17 software, SAS version 9.4, and multilevel analysis. To investigate the determinant factors, we applied multilevel statistical analysis.

**Results:**

Among 1,246 currently breastfed children aged 6–23 months, based on a 24-h recall method, it showed that 1,066 (85.56%), 550 (44.15%), 1,025 (82.24%), and 1,098 (88.09%) of children had inadequate minimum dietary frequency, minimum meal frequency, minimum milk feeding frequency, and minimum acceptable diet, respectively. The multilevel analysis revealed that individual-level factors, such as mothers with primary, secondary, and higher educational levels, middle and richest household wealth, children aged between 12–17 and 18–23 months, received a postnatal check, and having one and three antenatal care follow-up had lower odds of feeding their children with inadequate minimum acceptable diet than their counterparts. At the zonal community level, children residing in urban areas and children residing in zonal communities with a high literacy level were less likely to have inadequate minimum acceptable diet.

**Conclusion:**

There is a high magnitude of inadequate minimum acceptable diet intake among children aged 6–23 months in Ethiopia. Mother’s education, household wealth, marital status, number of families, age of child, postnatal check, community-level education, living in rural areas, and number of people under the age of 5 were significant factors of inadequate minimum acceptable diet. The findings highlight that, to increase the minimum acceptable diet intake in Ethiopia, policymakers and other stakeholders need to prioritize enhancing household wealth status and improving the accessibility of education.

## Introduction

Nutrition has a substantial impact on people’s health and is closely linked to social and cognitive development (1). A major contributing factor to the worldwide burden of disease is poor nutritional quality (2, 3). Infants’ dietary demands evolve quickly as they grow. Infants and young children still do not consume enough of the foods that make up a balanced diet, such as fruits, vegetables, whole grains, fibers, nuts, and seeds, and they consume too little free sugar, processed meat, sugary snacks, and beverages (3).

One of the eight basic indicators for evaluating infant and young child feeding (IYCF) practices proposed by the World Health Organization (WHO) was the minimum acceptable diet (MAD) for children 6–23 months old (4). It is the percentage of children between the ages of 6 and 23 months who eat a diet that is at least satisfactory. The MAD can be calculated as the sum of the minimal dietary diversity (MDD) and minimum meal frequency (MMF). A MAD should be provided to infants and young children to support proper growth and mental development (5). Moreover, consuming five or more of the eight suggested food groups indicates an MDD. The proportion of breastfed and non-breastfed children aged 6–23 months who consumed solid, semi-solid, or soft foods (as well as milk feeds for non-breastfed children) the required minimum number of times or more is known as the MMF (4).

Globally, one-third of the eight million childhood deaths that occur each year before the age of five are due to inadequate MAD (6). More importantly, the global prevalence of MAD was 19% (2, 3). Undernutrition and a lack of variety in diets are global public health issues, especially in low- and middle-income countries (LMICs) (7). Only one in four children aged 6–23 months in LMICs obtain the variety of food necessary for growth and development (8). Recent research from LMICs indicates that children’s MAD ranges from 6.1 to 36% (9). The countries of sub-Saharan Africa are most at risk from this global burden. In sub-Saharan African nations, it was estimated that 9.89% of children aged 6 to 23 months had MAD intake (1). Compared to the remaining regions, the percentage of children in East Africa who consume the MAD is only 11.56%. Notably, in Ethiopia, according to the 2019 Ethiopia Mini Demographic and Health Survey report, the national rates of stunting, underweight, and wasting among children under the age of 5 were, respectively, 37, 21, and 7%; these figures represent the impact of undernutrition in the first 1,000 days of life (5), which could be largely attributed to the county’s low MAD, where only 11.3% of children satisfied the standard (10, 11).

Less than one-fourth of infants aged between 6 and 23 months meet the standards for dietary diversity and feeding frequency that are suitable for their age, and few children in many countries receive supplemental foods that are safe and nutritionally adequate (11). Insufficient supplemental feeding is still common in many developing nations (9). Feeding a child a MAD ranges from 7% in Ethiopia to 36% in Nepal, according to recent demographic and health survey results of 10 Asian and African nations, including Ethiopia. This shows that feeding a child the minimal appropriate food is a serious issue both internationally and in developing nations (12). One of the biggest challenges to children’s physical development, mental development, and overall survival in the current world is consuming low-quality foods (13).

Inadequate MAD is still a flourishing health problem in developing countries, particularly in sub-Saharan Africa (14). Furthermore, inadequate MAD can lock an entire generation into stunted physical and intellectual development between the ages of 6 and 23 months, eroding human capital in poor countries. Without taking steps to address inadequate MAD feeding among newborn and young child, the health millennium development goals will not be met (2, 9). Even though Ethiopia has slightly achieved significant progress in reducing the proportion of children aged 6-23-month who are suffering from an inadequate MAD, the magnitude of the problem still is of great concern. During the survey periods of 2011, 2016, and 2019, 4.1, 7.3, and 11.3% of infants and young children in Ethiopia received the MADs, respectively (1). The health and development of children under the age of 2 years are influenced by low dietary diversity and MMF problems (15, 16).

Inadequate MAD remains one of the colossal obstacles to human wellbeing, affecting all areas of a child’s growth and development. Inadequate MAD during childhood can lead not only to long-term health problems but also to challenges in educational attendance and limited work opportunities in the future. Moreover, inadequate MAD feeding can also slow recovery from wounds and illnesses, and it can complicate diseases such as measles, pneumonia, malaria, and diarrhea, leaving the body more susceptible to disease (16, 17).

The immediate consequences of inadequate MAD during the early formative years include undernourishment, stunted growth, morbidity, unprecedented mortality, and delayed mental and motor development (9, 18). Inadequate MAD in the early stages of life can lower a child’s resistance to infections (13). Moreover, the potential negative impact of inadequate MAD feeding goes beyond the individual, affecting society and future generations (19). To the best of our knowledge, there is no latest study that examines the determinants of inadequate MAD feeding practices in Ethiopia using zones as a unit of analysis in accordance with the most recent WHO and UNICEF guidelines for assessing infant and young child feeding practices (4). Despite the fact that identifying the potential determinants and high-risk areas of infant and young child feeding practices will help to improve the nutrition status for child feeding.

Different studies were focused on the determinants of complementary feeding and inadequate MAD among 6–23-month-old children using classical models such as binary and multilevel logistic regression indices at the cluster level (20–22). A better understanding of where and why inadequate MAD occurs and the associated factors of inadequate MAD in Ethiopia is essential for designing strategies that will contribute to reducing the burden of inadequate MAD in Ethiopia. Exploring the significant factors of inadequate MAD intake in Ethiopia helps further understand where in a specific location the intake of inadequate MAD among children is most prevalent and supports the development of better nutritional interventions for policymakers.

Most of the previous researchers focused on multilevel analysis at the enumeration area (cluster level); however, this approach poses challenges for policymakers, implementation, and recommendation as enumeration areas are complex, tedious, and not well-defined, making it difficult to identify which cluster is at risk. Notably, our study uses the zone as a second-level (cluster-level) analysis. In Ethiopia, a zone is recognized by the whole population and has clear boundaries, a defined population, clear demarcations, and its own hierarchical administration. However, this is not the case for the enumeration areas. To address the above-stated gaps, this study aimed to explore the multilevel analysis of inadequate MAD feeding practices among infants and young children aged 6–23 months using the latest 2019 EMDHS.

## Methods

### Study area

**Figure 1:**
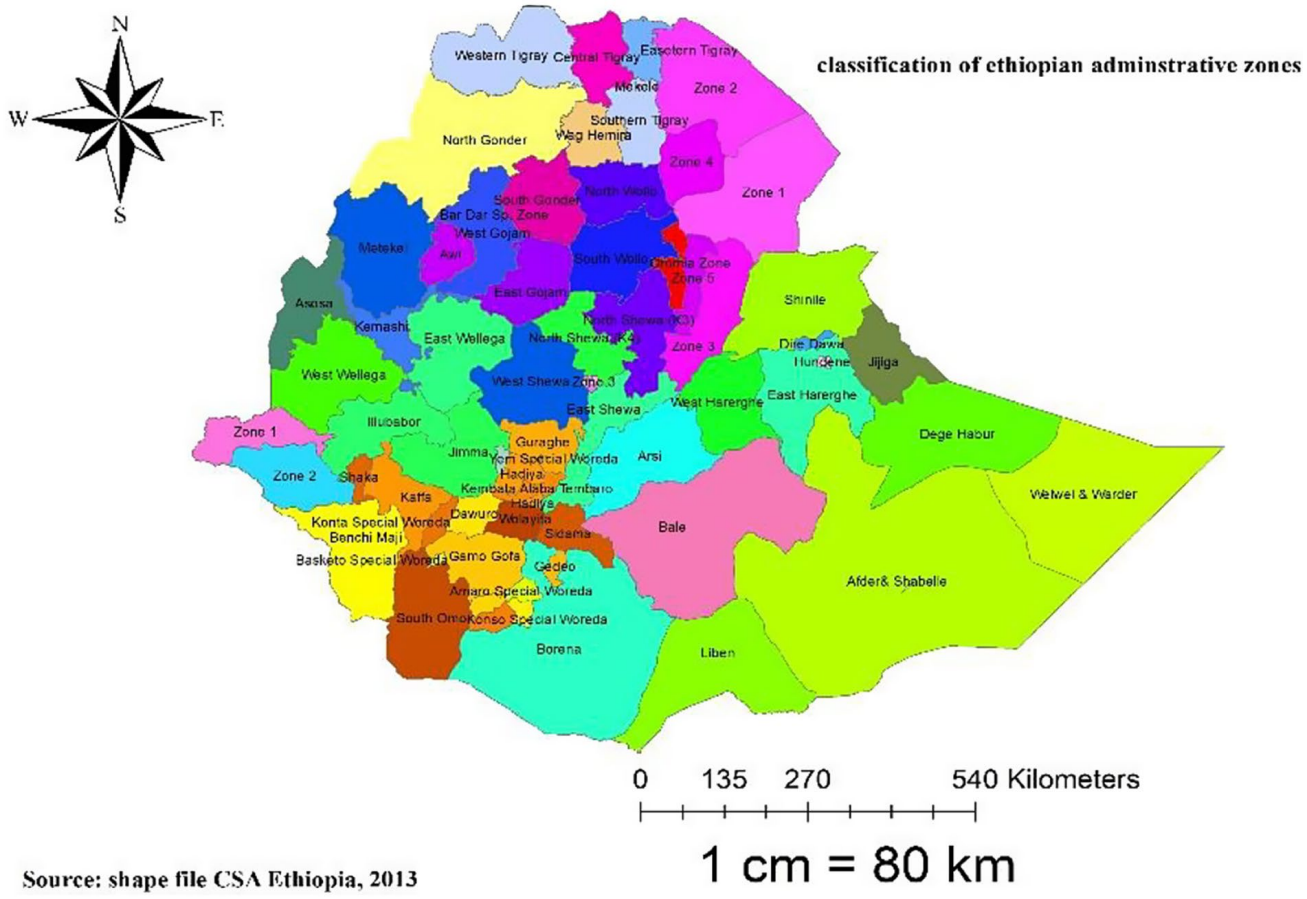
Map of Ethiopia with 72 administrative zones.

The study is Ethiopia, the oldest independent country in Africa, located on the Horn of Africa at 3° 14°N and 33° 48°E. The majority of Ethiopia’s political history has been monarchical, existing for over 2,000 years, dating back to the first century B.C. (23). Ethiopia is divided administratively into two city administrations and nine regional states. Following the division of regions into 72 zones, zones are further divided into 817 districts (weredas), 14,850 rural kebeles, and 1,478 urban kebeles (23, 24). Ethiopia is bordered by Eritrea to the north, Djibouti to the east and southeast, Somalia to the east, Kenya to the southwest, and Sudan to the west, with a total boundary length of 5,328 km. With an estimated population of more than 110 million, it is the second most populous nation in sub-Saharan Africa (23, 24) (Figure 1).

### Data sources and study design

To investigate the multilevel analysis of factors associated with inadequate MAD among Ethiopian children aged 6–23 months, we used a secondary analysis of the 2019 Ethiopia Mini Demographic and Health Survey (EMDHS). From 21 March 2019 to 28 June 2019, a community-based cross-sectional study was conducted in all regions of Ethiopia. Once authorization is obtained by writing a mini project from: https://dhsprogram.com, the DHS data can be freely accessed according to the procedures. Among the six record files, the researcher used children’s records or Kids Record (KR) files because this file contains information on women and children for this specific research, and important variables related to inadequate dietary diversity, MMF, and MAD were extracted from the dataset. Our study used a cross-sectional study design using data collected directly from a cross-sectional data source.

### Study population

For this study, the source population included all living children aged 6–23 months during the 5 years preceding the survey period across Ethiopia, whereas children aged 6–23 months living with their mother in the selected enumeration areas (EAs) or primary sampling units of the survey clusters were our study populations. The mother or caregiver was interviewed for the survey, and mothers who had more than one child during the 2 years before the survey were asked about the most recent child.

### Sample size and sampling procedure

**Figure 2:**
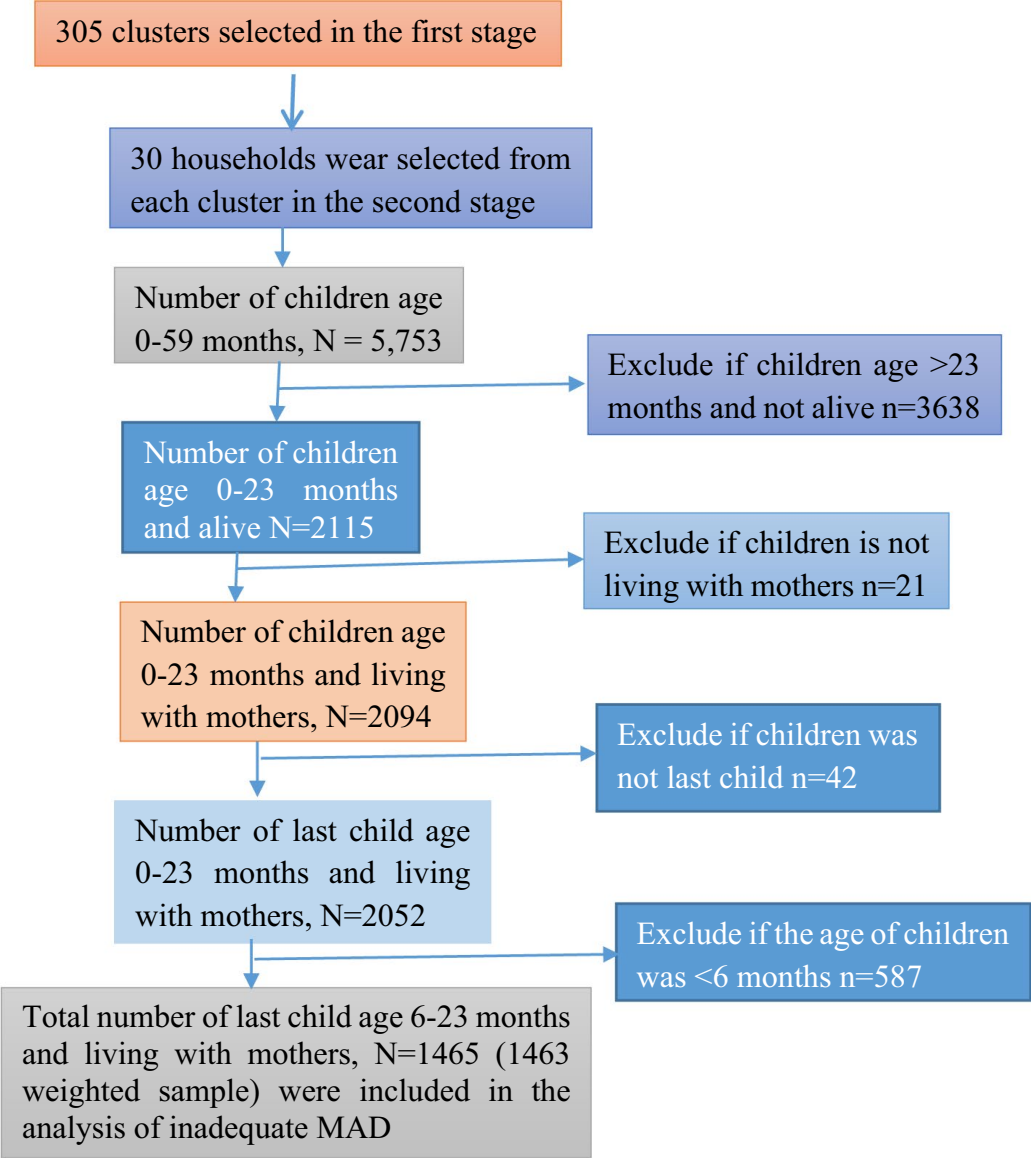
Flowchart of data extraction and sampling procedure.

The sample for the 2019 EMDHS was designed to provide estimates of key indicators for the country as a whole, for urban and rural areas separately, and for each of the nine regions and the two administrative cities (5). A stratified two-stage cluster sampling procedure was used to select the nationally representative sample. In the first stage, 305 EAs were chosen, with probabilities proportionate to EA size (93 in urban and 212 in rural areas) (based on the 2019 EPHC frame). Finally, a total weighted sample of 1,463 children in the age category of 6–23 months were included in this study (Figure 2).

### Inclusion and exclusion criteria

All children aged 6–23 months, preceding 5 years of the survey in the selected EAs, who were alive and the last child in the last 2 years living with the mother, were included in this study. However, children in the age category of 6–23 months who were not the last child, children aged >23 months, and children aged <6 moths were excluded.

### Study variables

#### Response variable

The outcome variable is the inadequate MAD of 6–23-month-old children. It is a binary outcome variable and coded as 0 if the child received adequate MAD and coded as 1 if the child did not receive inadequate MAD, which comprises children with the minimal possible requirements for dietary diversity and meal frequency in both breastfeeding and non-breastfeeding children. Operationally, MAD for currently breastfeeding children is defined as “receiving at least the MDD and MMF for their age during the previous day.” Similarly, MAD for children not currently breastfeeding is “receiving at least the MDD and MMF for their age during the previous day as well as at least two milk feeds.” Their mother was asked during the survey about the types and frequency of foods the child had consumed the day or night before the interview (25).

**Figure 3:**
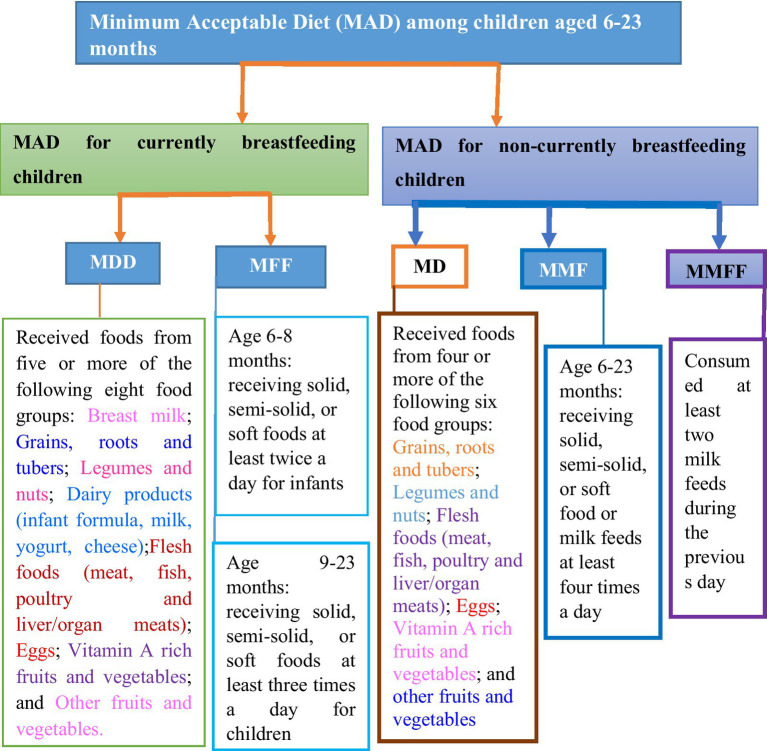
Diagrammatic representation of the definition of response variable.

According to the updated WHO 2021 IYCF indicators, breast milk is added as the eighth food group, and the cutoff for the minimum is increased to five food groups. If children aged 6–23 months feed five out of eight food groups during the day or night preceding the survey, the following food items are considered to have received MDD. The eight defined food groups are breast milk; grains, roots, and tubers; legumes and nuts; dairy products (including infant formula, milk, yogurt, and cheese); flesh foods (including meat, fish, poultry, and liver/organ meats); eggs; vitamin-A-rich fruits and vegetables; and other fruits and vegetables (4). MMF is defined as providing two or more times of solid, semi-solid, or soft feeds for breastfeeding children aged 6–8 months; three or more feeds for breastfeeding children aged 9–23 months; and four or more feeds for non-breastfeeding children aged 6–23 months. At least one of the four feeds must be a solid, semi-solid, or soft feed. Minimum milk feeding frequency for non-breastfed children aged 6–23 months (MMFF) is defined as the percentage of non-breastfed children who consumed at least two milk feeds during the previous day, which includes formula feed or any animal milk except human milk or semi-solid or fluid yogurt or fermented products made with animal milk (4) (Figure 3).

#### Independent variables

In this study, the independent variables were selected based on the various literature reviews and the availability of data in the 2019 EMDHS dataset. Most importantly, we checked multi-collinearity between variables using the variance inflation factor (VIF), and all variables had VIF values less than 10, which correlated with each other. This study considered variables identified in previous studies (9, 26–34) as the most related explanatory variables. Moreover, those explanatory variables were classified as individual-level variables if they were measured from the individual and as zonal community-level variables if they were measured from the zonal community.

If a woman was exposed to at least one of the three media sources (television, radio, or newspaper), she was considered to have media exposure; if not, she was considered to not have media exposure. The zonal community-level women’s education: At the national level, the median educational attainment was 5 years. As a result, the median value of the aggregated zonal clusters below 50% was categorized as having low education for women, and the median value of the aggregated zonal clusters 50% and higher was categorized as high education for women (10). The zonal community-level wealth index: The median values of the various household wealth categories were used to calculate the zonal community-level wealth index, which measures zonal community poverty. The aggregated zonal clusters were then divided into low and high categories (27, 28).

#### Statistical data analysis

The study used both descriptive and inferential statistics, and the sample weights were applied to compensate for unequal probabilities of selection across the geographically defined strata and to account for non-responses. Additionally, to obtain reliable estimates and standard errors, the data were weighted or standardized using the women’s sample weight. Frequencies and percentages were employed as descriptive measures to analyze information about the study participants’ characteristics. In this study, data extraction, recoding, and logistic analysis were calculated using STATA version 17 and SAS version 9.4.

#### Multilevel logistic regression analysis

In this study, we extended the binary logistic regression models by allowing random effects to accommodate correlations. The hierarchical nature of the DHS data, which violates the independence assumptions of the standard logistic regression model, was handled with a multilevel logistic regression analysis. Children in the same zone are more likely to be similar to each other than from another zone. This finding implies that there is a need to take into account the zonal variability by using advanced models such as the multilevel logistic regression model (35).

The multilevel process was stepwise. In this study, we followed Singer’s multilevel model development, and five models were constructed. First, an empty/intercept-only model, excluding independent variables, was constructed to assess the effect of zonal variation on the problem of children having inadequate MAD and to provide evidence to assess the random effect using the ICC value. Individual-level factors were included in the second model (Model II). In the third model (Model III), both random intercept and random slope models with level-1 predictors were incorporated. However, due to computational limitations, this model did not converge when all level-1 predictors were included as random slopes. As a result, we excluded the random coefficients except for mothers’ education and wealth index. Therefore, in this model, we only examined the influence (effect) of mothers’ educational level and wealth index between zones (allowing them to randomly vary between zones) with other fixed effects (by setting the variance of other coefficients to zero) on children having inadequate MAD. In the fourth model (Model IV), both individual-level and community-level factors were incorporated. To observe random effects (a measure of variation), we used the intraclass correlation coefficient (ICC) and the proportional change in variance (PCV). Stepwise variable selection was also employed in this study. Model comparisons were performed using AIC and BIC values, with the model having the lowest values of AIC and BIC considered the best fit. The Pearson Chi-squared statistic divided by the degree of freedom was used to asses fit of the models.

The robust model, which includes all interaction effects at time, is expressed as follows (Equation 1):


(1) $$\begin{array}{l} \text{logit}(\pi_{\mathrm{ij}})=\log(\frac{\pi_{\mathrm{ij}}}{1-\pi_{\mathrm{ij}}})=\gamma_{00}+{ϒ}_{\mathrm{ko}}X_{\mathrm{kij}}+{ϒ}_{0q}Z_{\mathrm{qj}}+\\ U_{0j}\;\text{to logit}(\pi_{\mathrm{ij}})=\log(\frac{\pi_{\mathrm{ij}}}{1-\pi_{\mathrm{ij}}})=\gamma_{00}+{ϒ}_{\mathrm{ko}}X_{\mathrm{kij}}+\\ {ϒ}_{0q}Z_{\mathrm{qj}}+U_{0j}\; \end{array}$$

## Results

### Results of descriptive statistics

**Figure 4:**
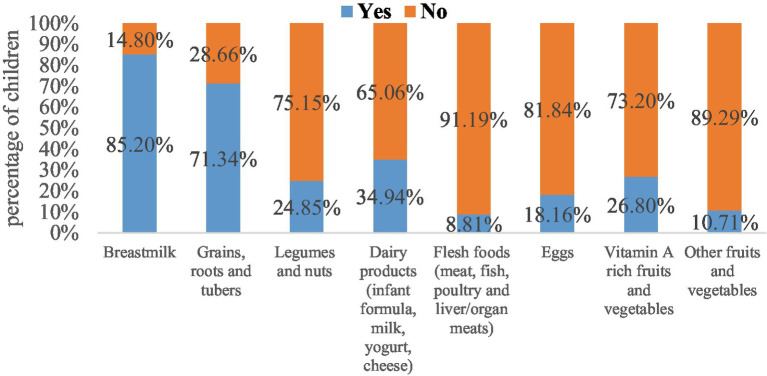
The magnitude of inadequate dietary intake across eight food groups among children aged 6–23 months in Ethiopia.

A total net weighted sample of 1,463 children aged 6–23 months was included in this study as a sample size. The majority of children, 1,246 (85.2%), were currently breastfed. The majority of children, 1,044 (71.3%), consumed grains, roots, and tubers, followed by 511 (34.9%) children who consumed dairy products (including infant formula, milk, yogurt, and cheese) in the past 24 h before data collection. However, smaller proportions of children, 157 (10.7%), consumed other fruits and vegetables, while 129 (8.8%) consumed flesh foods (meat, fish, poultry, and liver/organ meats) (Figure 4).

**Figure 5:**
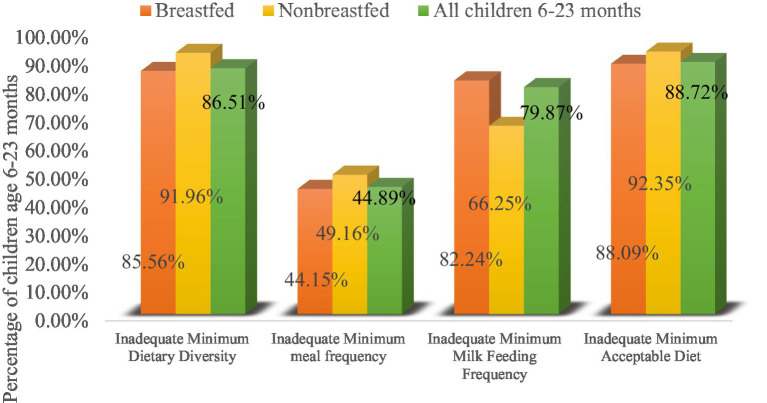
Percentage of children fed inadequate MDD, MMF, MMFF, and MAD with currently breastfeeding status.

Among the 1,246 currently breastfed children aged 6–23 months based on a 24-h recall method, it showed that 1,066 (85.56%), 550 (44.15%), 1,025 (82.24%), and 1,098 (88.09%) of children had inadequate MDD (i.e., not receiving the recommended minimum dietary diversity or not feeding five or more food items), MMF, MMFF, and MAD, respectively, as shown in (Figure 5, orange color). Figure 5 (gold color) shows that out of the 217 non-breastfed children aged between 6 and 23 months, 199 (91.96%) children did not meet the minimum dietary diversity criterion and slightly less than half of the children, while 106 (49.16%) did not meet the MMF criterion. Two-thirds (66.25%) had inadequate MMFF (i.e., not consuming at least two milk feeds during the previous day) and 200 (92.35%) of children had MAD for non-breastfed children only.

Overall, out of 1,463 children, the prevalence of inadequate MDD, MMF, and MMFF intake among children aged 6–23 months in Ethiopia was 86.51, 44.89, and 79.87%, respectively. The overall prevalence of inadequate MAD intake among children aged 6–23 months in Ethiopia was 1,298 (88.72%), with respect to all three IYCF practices (breastfeeding status, number of food groups, and times they were fed during the day or night before the survey) (Figure 5).

### Regional prevalence of inadequate MDD, MMF, and MAD

**Figure 6:**
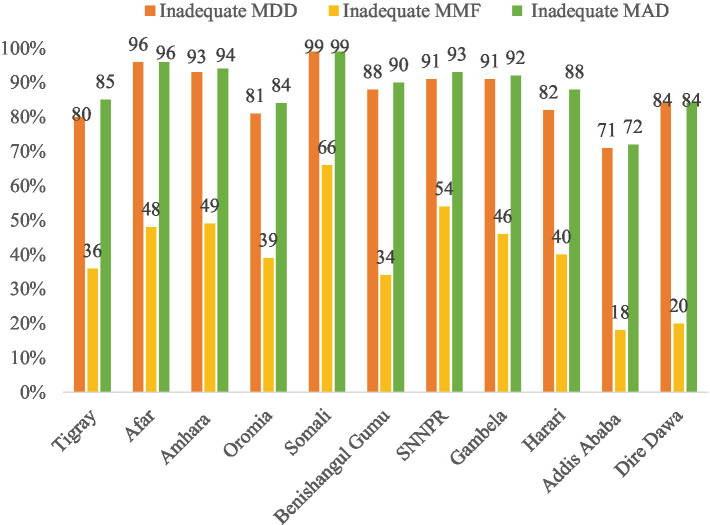
Prevalence of inadequate MDD, MMF, and MAD feeding practice across the Ethiopia region.

The prevalence of inadequate MDD, MMF, and MAD varies across the regions of the country. The highest prevalence of inadequate MDD and MMF were observed in Somali (98.8 and 65.9%, respectively), while the lowest prevalence was observed in Addis Ababa (70.9 and 17.7%, respectively). Similarly, the percentage of children who did not receive the recommended MAD was highest in Somali, Afar, and Amhara (98.8, 96.4, and 94.2%, respectively), and lowest in Addis Ababa (72.2%) (Figure 6).

### Sociodemographic characteristics of mothers or caregivers

Of the total number of mothers interviewed, one-half (725, 49.6%) of mothers/caregivers were found in the age group of 25–34 years, with a median age of 27 (IQR: 23, 30) years. Nearly two-thirds of women (944, 64.5%) had no media access, and among them, 859 (66.2%) of their children did not receive the recommended MAD. The majority of mothers were currently married (1,381, 94.4%). Among the total mothers, 609 (41.7%) had primary education and 539 (36.8%) were orthodox. Regarding the wealth index, approximately 604 (41.3%) mothers have a poor wealth index; among the children, 568 (43.8%) did not receive MAD. The chi-squared test of association indicates that inadequate MAD was significantly associated with categorical predictor variables, such as age of women, mother’s education, mother’s marital status, household wealth index, media exposure, household family size, toilet facility type, and current pregnancy of women (Table 1).

**Table 1:** Association of inadequate MAD with demographic and socioeconomic characteristics of study participants.

Independent variables	Categories	Total weighted frequency *n* (%)	Inadequate MAD, *n* (%)	*p*-value
Sex of household head	Male	1,259 (86.1)	1,119 (86.2)	0.7431
	Female	204 (13.9)	179 (13.8)	
Age of women (years)	15–24	473 (32.3)	419 (32.3)	0.0293
	25–34	725 (49.6)	642 (49.5)	
	35–49	265 (18.1)	237 (18.2)	
Mother’s education	No Education	650 (44.4)	602 (46.4)	<0.0001
	Primary	609 (41.7)	531 (40.9)	
	Secondary	120 (8.2)	97 (7.5)	
	Higher	84 (5.7)	68 (5.2)	
Marital status of mother	Unmarried	82 (5.6)	64 (4.9)	0.0013
	Married	1,381 (94.4)	1,234 (95.1)	
Household wealth index	Poor	604 (41.3)	568 (43.8)	<0.0001
	Middle	276 (18.8)	236 (18.2)	
	Rich	583 (39.9)	494 (38.0)	
Media exposure	No	944 (64.5)	859 (66.2)	0.0003
	Yes	519 (35.5)	439 (33.8)	
Household family size	≤5	805 (55.0)	698 (53.8)	0.0087
	6 and above	658 (45.0)	600 (46.2)	
Number of U5C	2 or less	1,315 (89.9)	1,162 (89.5)	0.1398
	3 and above	148 (10.1)	136 (10.5)	
Religion of respondents	Orthodox	539 (36.8)	476 (36.7)	0.1777
	Catholic	8 (0.6)	8 (0.6)	
	Protestant	413 (28.2)	371 (28.6)	
	Muslim	474 (32.4)	414 (31.9)	
	Traditional/other	29 (2.0)	29 (2.2)	
Toilet facility type	Improved	262 (17.9)	228 (17.6)	0.0001
	Unimproved	737 (50.4)	634 (48.8)	
	No facility	464 (31.7)	436 (33.6)	0.0582
Source of drinking water	Improved	981 (67.0)	859 (66.2)	
	Unimproved	482 (33.0)	439 (33.8)	
Currently pregnant	No or unsure	1,379 (94.3)	1,218 (93.8)	0.0387
	Yes	84 (5.7)	80 (6.2)	

### Health-related characteristics of mothers and child characteristics

Of the children, boys comprised 764 (52.2%) of the total. More than one-third (552, 37.7%) of the children were found in the age group from 12 to 17 months old with a median age of 14 (IQR: 10, 18) months. The majority (1,246, 85.2%) of children were currently breastfeeding. Of the total women, 661 (45.2%) had four and above ANC follow-up. More than half (806, 55.1%) of mothers delivered at health facilities. The majority (1,265, 86.5%) of women did not attend postnatal check within 2 months. The highest proportion of inadequate MAD feeding was observed among children aged 12–17 months (36.7%), while the lowest percentage was noted from the 18–23 months age group (29.1%), respectively. Overall, 47.2% of second-order to fourth-order births were not fed adequate MAD (Table 2).

**Table 2:** Association of MAD with health-related characteristics of mothers and child characteristics of study participants in Ethiopia.

Independent variables	Categories (codes)	Total weighted frequency *n* (%)	Inadequate MAD, *n* (%)	*p*-value
Sex of child	Male	764 (52.2)	676 (52.1)	0.7065
	Female	699 (47.8)	622 (47.9)	
Age of the child (months)	6–11 m	476 (32.5)	444 (34.2)	0.0006
	12–17 m	552 (37.7)	476 (36.7)	
	18–23 m	435 (29.8)	378 (29.1)	
Child is twin	No	1,448 (99.0)	1,283 (98.8)	0.1735
	Yes	15 (1.0)	15 (1.2)	
Breastfeeding status	No	217 (14.8)	200 (15.4)	0.0076
	Yes	1,246 (85.2)	1,098 (84.6)	
Birth order	First order	354 (24.2)	304 (23.4)	0.0015
	Second to fourth order	691 (47.2)	604 (46.5)	
	Fifth and above order	418 (28.6)	390 (30.1)	
Preceding birth interval	≤24 months	268 (18.3)	237 (18.3)	0.1302
	25–35 months	271 (18.5)	242 (18.6)	
	≥36 months	924 (63.2)	819 (63.1)	
Postnatal check	No	1,265 (86.5)	1,140 (87.8)	<0.0001
	Yes	198 (13.5)	158 (12.2)	
Place of delivery	Institution	806 (55.1)	699 (53.9)	0.0089
	Home	657 (44.9)	599 (46.1)	
Number of antenatal care visits	No visit	351 (24.0)	312 (24.0)	0.0110
	1–3 visits	451 (30.8)	415 (32.0)	
	4 and above visits	661 (45.2)	571 (44.0)	

### Zonal community-level characteristics

From the zonal community-level variables, the majority of respondents (1,050, 71.8%) were rural inhabitants, of whom 943 (72.7%) had inadequate MAD intake. More than half (798, 54.5%) of the mothers were from a zonal community with a high wealth index level. Nearly half of the mothers (737, 50.4%) were from the zonal community with low zonal community-level education. Of these, 53.7% of children did not receive adequate MAD (Table 3).

**Table 3:** Association of MAD with zonal community-level characteristics.

Independent variables	Categories	Total weighted frequency *n* (%)	Inadequate MAD, *n* (%)	*p*-value
Residence	Urban	413 (28.2)	355 (27.3)	0.0395
	Rural	1,050 (71.8)	943 (72.7)	
Community-level education	Low	737 (50.4)	697 (53.7)	<0.0001
	High	726 (49.6)	601 (46.3)	
Community-level wealth	Low	665 (45.5)	592 (45.6)	0.0116
	High	798 (54.5)	706 (54.4)	
Community-level media exposure	Low	737 (50.4)	683 (52.6)	<0.0001
	High	726 (49.6)	615 (47.4)	
Community-level ANC utilization	Low	859 (58.7)	781 (60.2)	0.3441
	High	604 (41.3)	517 (39.8)	

### Multilevel model parameter results

#### Random effect and model comparison

As shown in Table 4, the ICC in the null model was 0.4924, indicating that 49.2% of the total variability for inadequate MAD was attributable to the difference between zones in our study, resulting in 50.8% of the variability to be accounted for the individuals (children’s) or other unknown factors. The large proportion of the variability in inadequate MAD explained by the zones emphasizes the importance of accounting for the hierarchical structure of the data.

**Table 4:** Model comparison criteria on multilevel logistic models.

Parameter	Model I	Model II	Model III	Model IV	Model V
Variance	3.1921	2.7462	2.6424	2.2380	1.2635
ICC	0.4924				
PCV	Reference	0.1397	0.1722	0.2989	0.6042
Standard error	0.8594	0.8817	0.5310	0.7542	0.3674
Z-score	3.71	3.11	3.07	2.97	3.44
*p*-value	0.0001	0.0009	0.0017	0.0015	0.0003
Model comparison					
AIC	909.61	857.19	873.81	854.13	846.59
BIC	916.99	915.01	929.94	918.58	913.51
Pearson X2 /DF	0.79	0.84	0.84	0.86	0.88

ICC, intraclass correlation coefficient; PCV, percentage change in variation; Model I, empty model (without the predictors); Model II, adjusted for random intercept model with level-1 predictors; Model III, adjusted for random intercept and random slope model with level-1 predictors; Model IV, adjusted for level-2 predictors in addition to Model III.

Regarding model comparison/fitness, the model that includes a random intercept with an individual-level predictor, as well as a community-level predictor (Model V), was the best-fitted model for the data (based on lower AIC and BIC fit statistics) compared to the other fitted models and selected for further interpretation (Table 4). Furthermore, in this model (Model V), the zonal community-level residual goes down from 3.1921 to 1.2635, so that the zonal level $R^{2}\;$ becomes $\mathrm{PCV}=R^{2}=\frac{\sigma_{e|b}^{2}-\sigma_{e|m}^{2}}{\sigma_{e|b}^{2}}=\frac{3.1921-1.2635}{3.1921}$ = 0.6042. The proportion of explained variance at the zonal community level is approximately 60.4%. This finding indicates that individual, community-level predictors explained 60.4% of variability at the zonal level for inadequate MAD. The Wald value (Z = 3.44, and the *p*-value is 0.0003) indicates that the random effects were statistically significant (Table 4).

### Factors associated with inadequate MAD intake

The overall test of Model V shows that level-1 predictors (mother’s education, wealth index, marital status, number of families, age of child, postnatal check, and ANC visit) and level-2 predictors (community-level education, residence, and number of people under the age of 5 years) were statistically significant with the odds of inadequate MAD (Table 5).

**Table 5:** Parameter estimate for the selected model (Model V) of inadequate MAD.

Fixed effect	Estimate (95%CI) SE	Odds ratio (95% CI)
Intercept	2.9374 (2.4548, 3.42) 0.2462*	18.8667 (11.6441, 30.5694)
Mother’s education		
Primary	−0.5157 (−0.942, −0.089) 0.2176*	0.597 (0.39, 0.915)
Secondary	−0.9974 (−1.623, −0.372) 0.3193**	0.369 (0.197, 0.689)
Higher	−1.2689 (−2.021, −0.517) 0.3837**	0.281 (0.133, 0.596)
No education (ref)	0	1
Age of women in year		
25–34	−0.4599 (−0.943, 0.023) 0.2466	0.631 (0.389, 1.024)
35–49	−0.4399 (−1.148, 0.268) 0.3614	0.644 (0.317, 1.308)
15–24 (ref)	0	1
Wealth index		
Middle	−0.9239 (−1.525, −0.323) 0.3066**	0.397 (0.218, 0.724)
Rich	−1.4475 (−2.069, −0.826) 0.3169***	0.235 (0.126, 0.438)
Poor (ref)	0	1
Religion		
Orthodox	−3.3631 (−9.017, 2.29) 2.8844	0.035 (0, 9.878)
Catholic	0.5884 (−16.589, 17.766) 8.7642	1.801 (6.244×10^−8^, 5.196×10^7^)
Protestant	−2.8046 (−8.44,2.831) 2.8752	0.061 (0, 16.959)
Muslim	−3.3232 (−8.96, 2.32) 2.8782	0.036 (0.00013, 10.18)
Traditional/other (ref)	0	1
Marital status		
Unmarried	1.0659 (0.321, 1.811) 0.38**	2.903 (1.379, 6.115)
Married (ref)	0	1
Number of families size		
6 and above	0.5093 (0.051, 0.967) 0.2337*	1.664 (1.053, 2.631)
≤5 (ref)	0	1
Age of child		
12–17 m	−1.2007 (−1.717, −0.685) 0.2633***	0.301 (0.18, 0.504)
18–23 m	−0.7078 (−1.252, −0.164) 0.2774*	0.493 (0.286, 0.849)
6–11 m (ref)	0	1
Breastfeeding status		
Yes	−0.6051 (−1.253, 0.043) 0.3306	0.546 (0.286, 1.044)
No (ref)	0	1
Birth interval		
≤24 months	0.0253 (−0.53, 0.58) 0.2832	1.026 (0.589, 1.787)
25–35 months	0.1453 (−0.393, 0.684) 0.2747	1.156 (0.675, 1.981)
≥36 months (ref)	0	1
Postnatal check		
Yes	−0.8697 (−1.399, −0.341) 0.2698**	0.419 (0.247, 0.711)
No (ref)	0	1
Number of ANC visits		
1 and 3 visits	−1.0628 (−1.723, −0.402) 0.337**	0.345 (0.179, 0.669)
4 and above visits	−0.4717 (−1.107, 0.163) 0.3239	0.624 (0.331, 1.177)
No visit (ref)	0	1
Place of delivery		
Institution delivery	0.07839 (−0.479, 0.636) 0.2846	1.082 (0.619, 1.889)
Home delivery (ref)	0	1
Residence		
Urban	−1.1313 (−1.636, −0.626) 0.2577***	0.323 (0.195, 0.535)
Rural (ref)	0	1
Community-level education		
Higher	−1.5148 (−2.577, −0.452) 0.542**	0.22 (0.076, 0.636)
Lower (ref)	0	1
Community wealth index		
Higher	0.2609 (−0.7, 1.221) 0.4901	1.298 (0.497, 3.392)
Lower (ref)	0	1
Community media exposure		
Higher	0.1147 (−0.935, 1.165) 0.5357	1.122 (0.393, 3.206)
Lower (ref)	0	1
Random effect parameters		
Mother’s education	1.4776 (0.413, 2.542) 0.5433**	4.382 (1.511, 12.711)
Wealth index	1.4912 (0.444, 2.537) 0.5343**	4.442 (1.559, 12.66)

*** indicates *p*-value <0.001, ** indicates *p*-value <0.001 and * indicates *p*-value <0.05, SE, standard error; CI, confidence interval.

Infants and young children whose mothers had primary, secondary, and higher educational statuses were 0.6, 0.37, and 0.28 times less likely to intake inadequate MAD than children whose mothers had no formal education, respectively. The estimated odds of children receiving inadequate MAD decrease by a factor of 0.397 [OR = 0.397, 95% CI: (0.218, 0.724)] and 0.235 [OR = 0.235, 95% CI: (0.126, 0.438)] times when their mothers are from the middle and rich households compared to women who are from poor households, respectively.

The estimated odds of inadequate MAD among children from unmarried women were 2.9 times [OR = 2.903, 95% CI: (1.379, 6.115)] higher than those of children from married mothers. The estimated odds of having children with inadequate MAD intake were 66% more likely among households that have six or more family members compared to women who have five or fewer family members [OR = 1.664, 95% CI: (1.053, 2.631)]. The estimated odds of feeding a child with inadequate MAD were 0.3 times [OR = 0.301, 95% CI: (0.18, 0.504)] and 0.49 times [OR = 0.493, 95% CI: (0.286, 0.849)] lower among children aged between 12–17 and 18–23 months than children aged 6–11 months, respectively.

The estimated odds of receiving inadequate MAD were 0.42 times [OR = 0.419, 95% CI: (0.247, 0.711)] lower among children who received a postnatal check within 2 months of birth than among those with no postnatal check. The estimated odds of having inadequate MAD intake for the child were 0.345 times lower among mothers who had between one and three ANC follow-ups [OR = 0.345, 95% CI: (0.179, 0.669)] than mothers who had no visits.

Regarding the place of residence, the estimated odds of receiving inadequate MAD were 0.32 times [OR = 0.323, 95% CI: (0.195, 0.535)] less likely among urban residents than those residing in rural areas. Children from a zonal community with a high level of mothers’ education were 0.22 times [OR = 0.22, 95% CI: (0.076, 0.636)] less likely to consume inadequate MAD than children from a zonal community with a low level of mothers’ education level (Table 5).

## Discussion

This study aimed to determine inadequate MAD feeding practice and associated factors among infants and young children in Ethiopia. Accordingly, the prevalence of inadequate MAD feeding practices was 88.72%, which shows that children aged 6–23 months have inadequate access to MAD. This finding is in line with the research conducted in East Africa (88.44%) (9), Lesotho (88.7%), Equatorial Guinea (88.6%) (36), and Nigeria (89.0%) (37) and the report of EMDHS 2019 (5).

However, our study indicated that this percentage is higher than studies conducted in rural communities of Goncha district, North West Ethiopia (87.4%) (38), Myanmar (84%) (31), Northern Ghana (74.8%) (37), Nepal (69.9%) (39), and Sri Lanka (32%) (40). Additionally, it was lower than studies conducted in Dembecha, North West Ethiopia 91.4% (38), Congo (95.4%), Cameron (94.5%), Zimbabwe (91.9%), Gambia (91.7%), Burundi (91.2%), and Senegal (90%) (36). The possible justification might be due to differences in food cultures and study periods. This finding is explained by the fact that some societies are agriculturally dominant in child feeding, whereas others depend on animal products.

This study found that, as the age of children increases, the odds of having inadequate MAD decreases, which implies that the practice of adequate MAD significantly increases as the child’s age increases. As children grow older, they typically have more exposure to a variety of foods and may have better access to nutrition education, either at home or in school, increased autonomy in food choices, better cooking skills, or parental guidance improving over time. This finding is in agreement with the studies conducted in Nepal (33, 41) Ghana (37), Malawi (29), and Ethiopia (14, 18).

In this study, women who are currently not married were more likely to have children with inadequate access to MAD than those who were married. This is the same as the research conducted in Indonesia (42), India (43), sub-Saharan Africa (14), and Ethiopia (44), which might be due to the fact that, currently, unmarried women are single, widowed, or divorced and have no support from husbands and less support from families or communities, which causes poor infant-feeding practices.

According to this study, if mothers have one to three antenatal care visits (ANCs) during their pregnancy, their child has a lower chance of having inadequate MAD (has a higher chance of having MAD) than mothers who have no visit. This finding is in line with a study conducted in Ethiopia (11). This agreement might be due to effective nutrition education and counseling (often provided during ANC visits) that might contribute to a MAD.

In this study, children who lived in rural residences were more likely to have inadequate MAD. This finding is supported by previous studies conducted in Nepal (39), the Democratic Republic of Congo (20), sub-Saharan African countries (14), and some parts of Ethiopia, particularly the Bench Maji Zone (11). The reason for having a higher chance of using adequate MAD in urban residences might be the result of the cumulative effect of a series of more favorable conditions, including better socioeconomic and educational conditions, which in turn lead to better caring practices for children and their mothers.

In this study, we found that infants and young children from households with medium and high wealth indexes were less likely to receive inadequate MAD than those from households with the lowest wealth index. This finding is in line with studies conducted in the Philippines (22), the Democratic Republic of Congo (20), Tanzania (45), and the Chelia District, in Ethiopia (39). This agreement indicates that poorer households may lack the purchasing power to provide a sufficiently varied diet for their children.

This study revealed that the education level of mothers was significantly associated with inadequate MAD among children aged 6–23 months. The odds of receiving MAD were higher among mothers who attended primary, secondary, and higher educational levels than those who had no formal education. A similar finding was reported in studies conducted in Tanzania (45), East Africa (9), and Ethiopia (46). It indicates that educated mothers have better nutritional knowledge and are likely to have more assertiveness, a higher position within the household, and a greater ability to allocate household resources on their own than mothers with no schooling.

Mothers who had a postnatal check within 2 months were less likely to feed inadequate MAD than mothers who had no postnatal check within 2 months. This result is also in agreement with findings in the Democratic Republic of Congo (20), East African region and India (47, 48), Ghana and Tanzania (34, 49, 50), and Ethiopia (50, 51). This agreement could be because the health information or education they obtained during their postnatal check visit could be the reason.

Notably, we also found that children from communities with higher educational levels had higher odds of being fed a MAD than children from communities with lower educational levels. This finding is supported by studies conducted in sub-Saharan Africa (14) and East Africa (9). This may also be because educated mothers are more likely to have sufficient knowledge and understanding of the practices of child feeding, as they received lessons in school on child feeding, which would improve their comprehension of the value of child feeding.

## Conclusion

This study highlights a high prevalence of inadequate MAD feeding practices in Ethiopia, with significant variation across different zones. Approximately 60.42% of the variability at the zonal community level for inadequate MAD is explained by individual-level and zonal community-level variables. The multilevel analysis identified both individual and community-level factors associated with inadequate MAD feeding practices. At the individual level, significant factors include the mother’s educational level, household wealth status, marital status of women, number of family members, age of the child, postnatal check-ups, and antenatal care visits (1–3 visits). At the community level, factors such as community education levels and rural residence are significantly associated with inadequate MAD among children aged 6–23 months.

These findings can aid policymakers in revising programs to achieve Sustainable Development Goals (SDGs) related to universal infant and young child nutrition coverage. To reduce inadequate MAD, an integrated approach involving multiple sectors is necessary, focusing on modifiable socioeconomic factors like women’s education and household wealth. For the Ethiopian Ministry of Health, it is recommended to promote antenatal and postnatal care visits in line with WHO recommendations, design public health interventions targeting higher-risk children, particularly those from the poorest households, and provide targeted child nutrition and feeding guidance for mothers, especially those with lower education levels. Additionally, promoting MAD feeding through social and behavioral change interventions is crucial for children aged 6–12 months, those in large households, rural residences, and children of unmarried mothers.

The Ethiopian Ministry of Education should enhance the accessibility of education and increase the number of educated mothers. The government should work toward improving the country’s economy and enhancing household wealth. Non-governmental organizations can use these findings to identify high-risk areas for potential investment in addressing inadequate MAD. Researchers should conduct further studies to explore why inadequate MAD remains a challenge in Ethiopia.

### Strength of study

- EDHS data, which are nationally representative of all regions of Ethiopia.
- Sampling weights to ensure the representativeness and standardize the sample.
- Multilevel modeling to accommodate the hierarchical nature of the EDHS.

### Limitations of the study

- One of the main limitations of this study was that it may not accurately reflect children’s past feeding experiences since it considers only 24-h feed.
- Another important limitation is that virtually all information collected in DHS surveys is subject to reporting and recall biases.
- Moreover, this study may be affected by a small sample size effect because the DHS data for this age specification with an issue is limited.
- The limitations also include lack of some variables and the presence of missing values in the Mini Demographic and Health Surveys (MDHSs) as well as reporting and recall biases.

## Data Availability

Publicly available datasets were analyzed in this study. These data can be found at: https://dhsprogram.com/data/.

## Glossary

AIC: Akaike’s information criterion
ANC: Antenatal care
AOR: Adjusted odds ratio
BIC: Bayesian information criterion
CSA: Central statistical agency
DHS: Demographic health survey
EA: Enumeration area
EMDHS: Ethiopia Mini Demographic and Health Survey
EPHC: Ethiopian population and housing census
EPHI: Ethiopia Public Health Institute
ICC: Intra class correlation
ICF: Intermediate care facility
IYCF: Infant and young child feeding
LMM: Linear mixed model
MAD: Minimum acceptable diet
MDD: Minimum dietary frequency
MLE: Maximum likelihood estimation
MMF: Minimum meal frequency
MMFF: Minimum milk feeding frequency
NNS: National Nutrition Survey
PCV: Proportional change in variance
SNNPR: South Nations Nationalities and Peoples Region
TIBF: Timely introduction to breastfeeding
USAID: United States Agency for International Development
WHO: World Health Organization
